# Development and application of new methods in micro-crystal Electron Diffraction (microED)

**DOI:** 10.1063/4.0001067

**Published:** 2025-10-27

**Authors:** William J

**Affiliations:** Exelixis, Alameda, CA, USA

## Abstract

MicroED is a diffraction-based EM technic usually restricted to small crystal a few microns thick, especially popular in the realm of small molecules. Because of the relative recency of this technic, tools and methods still need to be developed to streamline the process and democratize the method. Combining fluorescence light microscopy, and plasma FIB milling with microED now allows to target and process protein crystals directly in their growth medium, alleviating the need to blot and further manipulate those delicate crystals. Furthermore, recent developments in microscope automation and image feature recognition methods allow to streamline microED data acquisition and processing now making it a high throughput method, in the realm of protein and small molecule crystals.

